# Stress, Coping Strategies and Related Factors in a Sample of Iranian Adolescents

**DOI:** 10.5812/kowsar.20741804.2241

**Published:** 2011-09-15

**Authors:** M Saffari, F Ghofranipour, M Mahmoudi, A Montazeri

**Affiliations:** 1 Department of Health Education, Tarbiat Modares University, Tehran, Iran; 2 Department of Epidemiology and Biostatistics, Tehran University of Medical Sciences, Tehran, Iran; 3 Department of Mental Health, Institute for Health Sciences Research, ACECR, Tehran, Iran

**Keywords:** Perceived stress, Adolescents, Coping, Iran

## Abstract

**Background:**

Since the stress and coping are the most reported problems by adolescents, this study aims to apprise the stressors, coping strategies and influential factors among male adolescents.

**Methods:**

The effect of stressors, coping strategies and some demographics variables were studied in 402 male adolescents in Tehran, Iran. A self-administered questionnaire was used to collect the data. A multiple regression was performed to assess factors related to perceived stress.

**Results:**

The mean age of adolescents was 15.44 (SD=0.68) years. The mean value of perceived stress for the whole sample was 17.99 (SD=6.02). Cognitive/emotional coping was the most frequent coping style. The findings revealed that there was a significant correlation between perceived and accumulative stress. In multiple regression analysis, the accumulative stress, social resources, parent`s education and grade point average were found to be significant predictors of perceived stress.

**Conclusion:**

The findings suggest that increased level of perceived stress is associated with accumulative stress, social resources and parent`s education that are modifiable factors. Stress management education might be a solution to overcome increased perceived stress.

## Introduction

Adolescence is a sensitive period in which person faces some biological changes associated with puberty, increased pressure for social integrity, challenges of new roles, future adulthood related stressors, conflicts with peers and so other problems that can produce stress.[1] Forty to 70 percent of adolescents experience one or more stressful events during the teenage period.[2] According to Lazarus and Folkman, stress is defined as a relationship between the person and the environment that is appraised by the person as relevant to his or her well-being and in which the person’s resources are taxed or exceeded.[3]

An individual will only perceive a situation as stressful if it is an issue for this person and if this person feels that it threatens or surpasses his/her internal or external resources.[4] Depending on how an individual perceives the situation, he/she will choose certain coping strategies (CS) over others. These strategies may facilitate adaptation at a later stage.[3][4]

Adolescents more readily tend to engage in experiences or encounter situations that are stressful and associated with developing emotional and behavioral problems. Teenagers are also at the stage of developing their personal styles of coping. The CS can be reviewed, modified if needed and crystallized from one experience of using certain mechanisms of coping to another, during adolescent years.[5][6] Perceptions of stress and coping responses have an embedded cultural component of values[7] which bring to question the cultural universality of models of coping that were validated predominantly on urban populations.

In the Midwest of the United States, economic decline has been found to have an adverse impact on adolescent mental health though moderated to a large extent by socioeconomic conditions, family functioning and parenting practices.[8][9] There have been few studies, albeit some speculation, on the impact of urban stressors such as low socioeconomic level, crowding, and poverty especially in developing countries on adolescents. However, the One in five of worldwide population are adolescent between 10 to 19 years old and developing countries comprised 85% of them.[10] In Iran as a developing country, approximately 1/3 of population are adolescents.[11] In the present study, we studied adolescents from economically depressed community of this country. On other hand, fewer studies have considered how demographic factors, such as economic status, mothers and fathers’ education, may influence adolescent functioning in developing countries.

There are qualitative differences between females and males in how adolescents respond to stress.[1] But most studies have investigated the differences between two genders and few studies have considered the type of stressor and CS and related factors in one of the two genders.

Thus, the aims of the present study were to understand stress and coping styles and investigate correlations between demographic characteristics of male adolescents and their perceived stress (PS) in a sample of Iranian adolescents.

##  Materials and Methods

Four hundred and two adolescents from 8 high schools that were located in 4 different zones in southern area of Tehran participated in the study. All participants were from grades 9 (Mean=15.44 years, S.D=0.68 years) and all were male. The multistage sampling method was used for selecting subjects. Cohen tables were used for computing sample size. Participants were recruited through schools using consent forms that were distributed and collected by researchers. Participation was voluntary. Ethical approval for the study was granted by the Tarbiat Modares University` institutional review board. The researcher developed SASCP based on extensive literature reviews of adolescent coping inventories across different cultural contexts. Guided by the transactional theoretical framework, the researcher included measures of accumulative stress (AS), (number and severity of problems), major stressors, affective responses, coping strategies (types and efficacy of them), and social resources (SR), (types and helpfulness). SASCP contains 10 questions that encompass the cognitive, affective, behavioral and social dimensions of stress and coping processes. This uses a 5 point likert scale for measuring severity, efficacy and helpfulness of related items. The SASCP has been found to be reliable, producing test–retest coefficient of 0.87 over 2 weeks. Internal consistency was α=0.82. PSS developed by Cohen, Kamarck, and Mermelstein (1983), measures one's perception of stress related to daily life. The PSS is based on the rationale that one's perception of stressful life events has a more profound effect on one's health than the actual event itself. In line with the transactional model of stress, the PSS asks general questions about the last month of time. The scale consists of 10 items that are answered on a 5-point Likert Scale ranging from 0-5 (never to very often). Test–retest coefficient of this scale was 0.83 and its Cronbach alpha for internal consistency was 0.76. The survey included a one-page questionnaire requesting demographic information, such as age, birth place, duration of residence, the number of family members living in the home, having siblings, parent`s education and job, and past semester grade point average. Adolescents were asked to complete measures during class time supervised by trained researchers. Descriptive statistical methods were used for the data analysis. Moreover, a pearson correlation analysis was used to assess variable's correlation. Multiple linear regression analysis was conducted to identify factors related to perceived stress. All statistical tests were conducted by SPSS software (Version 17, Chicago, IL, USA).

## Results

The mean age of the adolescents was 15.44±0.68 years. Most of them were born in Tehran (74.9%) and 94.5% of them were living with both parents. Most fathers were self-employed (60%) and had secondary level education (54.5%). Most mothers were housekeeper (96%) and had secondary level education (55.5%). Past semester, grade point average of the participants was 14.91±2.41 (from 20) and 61.2% of the adolescents were living in a private house. The demographic characteristics of the adolescents were shown in Table 1.

**Table 1:** Sample characteristics.

	**No (%)**
Age (year; Mean, SD)	15.44 (0.687)
Birth place	
Tehran	301 (74.9)
Other	101 (25.1)
Duration of Residence in Tehran (year; Mean, SD)	14.27 (2.85)
Number of Family members	
≤3	36 (9)
4	154 (38.3)
5≥	212 (52.7)
Living with...	
Parents	380 (94.5)
One parent	19 (4.7)
Relatives	3 (0.7)
Having siblings	
Yes	376 (93.5)
No	26 (6.5)
Father’s job	
unemployed	14 (3.5)
officer	73 (18.2)
worker	74 (18.4)
other	241 (60)
Mother’s job	
housekeeper	386 (96)
officer	10 (2.5)
worker	6 (1.5)
Father’s education	
Higher	32 (8)
Secondary	219 (54.5)
Primary	151 (37.6)
Mother’s education	
Higher	17 (4.2)
Secondary	223 (55.5)
Primary	162 (40.3)
Past semester grade point average (0-20; Mean, SD)	14.91 (2.41)
Housing type	
Landlord	246 (61.2)
Tenant	145 (36.1)
Other	11 (2.7)

The more frequent problems that were reported in AS section were worries about the future, e.g., college, choosing a major, finding a job (301, 74.9%); academic related concerns, e.g., exams, grades, schoolwork (223, 55.5%) and financial problems (213, 53%). The biggest problem in the view of participants that they facing it in the past 3 month were worries about the future (141, 35.1%). academic (105, 26.1%) and financial (54, 13.4%) problems that were in next orders respectively. The severity of this problem was reported as very severe (108, 26.9%), severe (113, 28.1%), relatively severe (130, 32.3%) and partially severe (51, 14.4%). 80.6% of participants reported it predictable and 90% of participants believed that the biggest problem was changeable and 83.1% of them believed that for the problem, they could find people or resources in their environment to help them to deal with it. Also 89.1% believed to have the ability or skills to solve the biggest problems. More frequent feelings when facing this problem were nervousness (246, 61%), fear (120, 32.3%), and sadness (67, 16.6%).

These CS were reported by participants, frequently: keeping people from knowing their problems (144, 35.8%), trying to see the positive site (129, 32.1%), hoping that a miracle would happen and telling themselves not to think or worry too much (both 97, 24.1%), avoiding things or people that may upset them (87, 21.6%), imaging something funny or thinking about something else and engaging in physical activities (both 79, 19.7%). With regard to classification CS to 3 classes of avoiding stressor, cognitive/emotional management and problem solving, 228 (56.7%) of participants selected some cognitive/emotional copings, 216 (53.7%) selected some avoiding copings and 173(43%) selected some problem solving coping strategies. The mean of perceived stress score that was measured by PSS was 17.99±6.02.

Pearson correlation test showed significant relationships between AS and PS; AS and CS; SR, AS and CS (Table 2). Results arose from multiple regression analysis (Table 3) showed that accumulative stress and social resources significantly predicted PS (p<0.01). However, parent`s education, and past semester average significantly predicted the perceived stress (p< 0.05).

**Table 2:** Relationships between the perceived stress, Accumulative stress, Coping strategies and Social resources.

	**1**	**2**	**3**	**4**
1. Perceived stress	-			
2. Accumulative stress	0.203^a^	-		
3. Coping strategies	0.066	0.163^a^	-	
4. Social resources	-0.089	0.136^a^	0.244^a^	-

^a^ Correlation is significant at the 0.01 level (2-tailed).

**Table 3:** Multiple regression analyses of perceived stress score against some sociodemographic and related factors.

	**B**	**β**	**CI**	
			**Lower **	**Upper**
Accumulative stress	0.038^b^	0.158	0.014	0.062
Coping strategies	0.006	0.044	-0.008	0.020
Social resources	-0.029 b	-0.144	-0.051	-0.008
Age	-0.491	-0.058	-1.366	0.383
Father`s education	0.734 a	0.142	0.121	1.346
Mother`s education	-0.641 a	-0.123	-1.260	-0.021
Siblings number	0.319	0.014	-1.987	2.625
Residence time	-0.032	-0.016	-0.240	0.176
Monthly expenses	9.159	0.043	0.000	0.000
Past semester grade point average	-0.295 a	-0.121	-0.553	-0.037

^a^ P<0.05,

^b^ P<0.01, R2 = 0.273, Adjusted R2 = 0.248, β, Standardized regression coefficients; B, Unstandardized regression coefficients; CI, Confidence interval (95%).

## Discussion

Despite that there has been a great deal of measurement construction researches on stress appraisal but little research has been conducted on adolescents.[12] This study describes PS and their associations with number and severity of problems, CS and SR in a sample of adolescents from southern area of Tehran. The major findings of the study are discussed below.

The study showed that worries about the future and academic related concerns were the most frequent reported problems that adolescent in this study were exposed to them. Other studies found similar findings about academic concerns.[13][14] Cognitive/emotional and avoiding coping styles were more practiced by participants. This revealed that they were not oriented to problem solving coping strategies or they did not know how to use them. This finding reveals necessity of the stress management techniques for education in adolescents.

We can see this finding in the way that under stress adolescents may not feel like mixing and socializing with others. They may not want to discuss their problems with others and in their attempt to be alone; they keep themselves away from others as much as possible. During this transitional period, the easiest strategy seems to be denial of the stressful event or deal with it by an emotional response. This finding is in consensus with Kausar and Munir’s research in which they found that adolescents who reported financial stresses used significantly less active coping and frequently used strategies that focused on externalizing feelings.[13] Using emotional coping was frequently reported in Zimbabwean adolescents too.[15]

The current study found that the increased PS in adolescents was associated with increased AS. It was not surprising, because the more problems, the more stress develops. However, the PS has a reverse relation to SR that showed the lower SR is related to a higher level of stress. These findings are consistent with Magaya et al. and Hieu and Thao studies.[15][16]

The study showed that there was an association between parent`s education and PS. Father`s education had a negative association with PS. While mother` s education had a positive significant association with PS. In Iran, it is mainly the father who provides financial support to the family and mother usually takes up role of a housewife who looks after the household chores and child rearing.

In the country, boys are subjected to special attitude from family and culture for being a boy. The values, standards and expectations for boys and girls are different. Presence of mother can be a support for a boy. In Iran which is a relatively patriarchal and yet emotional society, boys feel more attached to their mothers. Therefore it is expected that more education in mothers can guide child how to cope with stress.

On other hand, fathers in a depressed socioeconomic society that are forced to provide adequate financial source, they work hard, have lower contact to children and could not provide enough support to children.

The boys usually model their fathers and in general higher educated fathers have more work responsibilities and then more work stress noticed by children may convey the stress to them. Regarding lower past semester average and more PS, one can discuss that the adolescents with high academic achievements know better to cope with stress and therefore lower their stress feelings. This relation has been observed in some other studies too.[17][18]

There are a number of methodological and conceptual limitations that should be noted. First, the participants used in this study were a sample of low socio- economic status (SES). Therefore, it was not possible to know if the factor structure generalizes to high SES groups. Second, we did not the two genders and we could not compare the differences between the two groups of adolescents. Finally, we chose our sample from one grade and the effect of age changes could not be shown and measured specifically.

In conclusion, increased level of PS is associated with AS, SR and parent`s education that are modifiable factors. Experience of being male and special types of stressors resulted in differential use of CS by male adolescents and relation between demographics and stress should be considered. However, findings from this study need to be interpreted with caution for the culture based nature of stress.

Moreover, the sample was recruited from governmental schools only, which in Iran are attended mainly by children from lower socioeconomic status and it does limit generalization of the findings to those attending private schools or those coming from other socioeconomic background. Finally it seems that stress management programs might be a solution to overcome increased PS in adolescents and a larger study to identify other factors is also recommended.
